# Characteristics of and Outcomes in COVID-19 Critical Care Patients in the Qassim Region, Saudi Arabia

**DOI:** 10.7759/cureus.48323

**Published:** 2023-11-05

**Authors:** Ruwayda M Alharbi, Manal A Selim, Abdullah A Alowayed, Waleed H Elhassan

**Affiliations:** 1 Department of Pharmacy Practice, College of Pharmacy, Qassim University, Buraydah, SAU; 2 Department of Pharmaceutical Care, Al Rass General Hospital, Ar Rass, SAU; 3 Department of Internal Medicine, King Saud Hospital, Unaizah, SAU

**Keywords:** infectious disease, severe acute respiratory syndrome, saudi arabia, intensive care unit, covid-19

## Abstract

Background and objective

Characterizing the epidemiological features of coronavirus disease 2019 (COVID-19) is highly important for developing and implementing effective control measures against it. However, there is scarce data about the presenting features and outcomes in ICU patients with COVID-19 in the Kingdom of Saudi Arabia (KSA). In light of this, this study aimed to assess the characteristics of and outcomes in COVID-19 ICU patients in KSA in order to describe and identify the risks associated with morbidity and mortality among them.

Methodology

A retrospective, hospital-based study was conducted from March 2020 to October 2021, which involved the review of medical records of the patients admitted to the ICU at COVID-19 treatment centers. The demographic data, comorbidities, signs, and symptoms of the patients were collected, along with data on the need for ventilation, duration of ICU stay, and fatality rate. All data were analyzed and the associations between variables were evaluated.

Results

A total of 172 patients were included in the study, most of them males (n=97, 56.4%) and elderly (69.6 ±18.2 years). The majority were Saudi nationals (n=143, 83.1%). Regarding comorbidities accompanying COVID-19, about 95 (55.2%) patients had cardiac diseases while 85 (49.4%) had diabetes; 33.7% of the patients needed mechanical ventilation versus 40.7% who needed non-mechanical ventilation. Significant associations were found in terms of age, comorbidities, and mortality rate (90, 52.3%), especially with cardiac diseases (p=0.025), diabetes (p=0.009), and kidney diseases (p=0.003).

Conclusion

COVID-19 infection is associated with a wide range of characteristics and outcomes. Raising awareness about the risk factors associated with COVID-19 infection will improve clinical outcomes by ensuring correct resource allocations and implementation of appropriate preventive measures.

## Introduction

In December 2019, an outbreak of severe acute respiratory syndrome (SARS) attributed to a new strain of coronavirus, specifically, severe acute respiratory syndrome coronavirus 2 (SARS-CoV-2), was identified in the Wuhan region of China. This disease, which was eventually termed coronavirus disease 2019 (COVID-19), is characterized by a flu-like syndrome, with features such as fever, myalgia, cough, and gastrointestinal symptoms. Most cases are mild in nature, and some might be asymptomatic; however, approximately 15% of patients have a more severe presentation, and 5% experience a critical disease [1]. In March 2020, COVID-19 was declared a global pandemic by the World Health Organization (WHO), and instances of the infection soon surpassed more than three million worldwide accompanied by expanding mortality rates [2]. The first case of COVID-19 in the Kingdom of Saudi Arabia (KSA) was reported on March 2, 2020, and the peak of the pandemic was reported in June and July 2020, with 4,919 being the highest number of cases recorded in a single day: on June 18, 2020 [3].

Patients with SARS-CoV-2 pneumonia have a high mortality rate in the ICU, which exerts significant pressure on hospital intensive care resources [4]. Because COVID-19 cases have increased exponentially around the world since the start of the pandemic, there is a need to document the temporal changes in patient characteristics over time, as well as the impact of real-world clinical practice on outcomes including ICU admissions, acute respiratory distress syndrome (ARDS)/respiratory failure, invasive mechanical ventilation (IMV), and all-cause mortality over time among patients with COVID-19 [5]. Acute hypoxemic respiratory failure progressing to ARDS is the most common presentation of COVID-19 infection in the ICU [6].

COVID-19 infection's severity and outcome are determined by both the virus and the host's immune response. Several studies from China, Italy, Sweden, and the United States have linked the disease's poor prognosis with advanced age, male gender, and pre-existing chronic conditions such as high blood pressure, cardiovascular disease, and diabetes. Simultaneously, pediatric cases have been reported to have a milder clinical illness course [7]. In Japan, an efficient healthcare system, with universal health insurance and the implementation of good personal hygiene practices such as the regular use of masks, as well as a very advanced level of medical practice and care, may have contributed to less severe outcomes related to COVID-19 in Japanese patients [8].

Given the high prevalence of hospitalization among COVID-19 patients in KSA, it is necessary to investigate if any distinct sociodemographic characteristics (e.g., age, sex) or medical factors [e.g., mandatory minute ventilation (MMV) usage, ICU admission] are linked with the increase in the morbidity and mortality risk in the country. Furthermore, the cost of hospitalization must be estimated [9]. To handle the pandemic effectively and efficiently, it is critical to provide government officials and physicians with clear guidance regarding risk factors, mortality, and how to prioritize screening, testing, isolation, or quarantining of COVID-19 patients [9]. In light of this, this study aimed to assess the characteristics of and outcomes in COVID-19 patients who developed critical illnesses requiring admission to ICU, with a view to describe and identify the risk factors associated with morbidity and mortality among them.

The abstract of this paper was presented at the Dubai International Pharmaceutical & Technologies Conference & Exhibition (DUPHAT) on January 10-12, 2023 (Identification Number: 112) [10].

## Materials and methods

Study design and population

A retrospective, hospital-based cohort study was conducted at the King Fahad Specialist Hospital and Al-Rass General Hospital, Qassim Region, KSA, during the period from March 2020 to October 2021. It involved adult patients who were admitted to the hospital with severe COVID-19 infection requiring ICU admission.

Inclusion and exclusion criteria

Adult patients with confirmed diagnosis of COVID-19 who had severe symptoms requiring admission to ICU were included in the study. COVID-19 patients with mild or moderate symptoms requiring hospitalization but not ICU admission, patients aged less than 18 years, COVID-19 patients admitted to ICU for any other reason, and patients with incomplete data in their medical records were excluded. The sample size of our study was 172 patients.

Data collection tool

The data were collected from the medical records of COVID-19 patients who were admitted to the ICU of King Fahad Specialist Hospital and Al-Rass General Hospital, Qassim Region, KSA. The data collection form included the following four parts: (1) the demographic data of the patients such as age, sex, and nationality (Saudi or non-Saudi); (2) comorbidities among the patients (cardiac disease, pulmonary disease, kidney disease, diabetes, obesity, immune diseases, cancer, transplantation history); comorbidities were classified into none, one to two, three to four, and more than four; (3) the signs and symptoms such as fever, cough, respiratory distress, gastrointestinal symptoms, oxygen saturation, and the need for invasive or noninvasive ventilation; and (4) data on the date of admission, discharge, or death.

Data analysis

The data was analyzed using SPSS Statistics version 26 (IBM Corp., Armonk, NY). All variables were assessed using descriptive statistics (percentages and frequencies). To determine any statistically significant relationships, associations between variables were identified using a one-way ANOVA test for scale parameters while a Chi-squared test was used for categorical parameters. A p-value ≤0.05 was considered statistically significant.

Ethical considerations

Ethical approval was obtained from the Qassim Region Research Ethics Committee (QREC, no: 1442-810574), and it was ensured that strict confidentiality was maintained and no recognizable information regarding patient identity was documented.

## Results

Table 1 presents the demographic data, symptoms, and comorbidities among the study participants (n=172). The mean age of the patients was 63.6 ±20 years and most of them were male (n=97, 56.4%) and Saudi nationals (n=143, 83.1%). Concerning the vital signs and symptoms of infection among patients, most of them had a cough (n=140, 81.4%), some had respiratory distress (n=49, 28.5%), and some had gastrointestinal symptoms (n=48, 27.9%); 54 (31.3%) patients had low oxygen saturation and 35 (20.3%) had a fever. Regarding comorbidities among patients, many of them had three to four comorbid diseases (n=67, 39.0%), while most of them had cardiac diseases (n=95, 55.2%), followed by diabetes in 85 (49.4%), and obesity in 56 (32.6%) patients.

**Table 1:** Demographic data along with symptoms and comorbid diseases among the study participants (n=172) SD: standard deviation

Characteristics	Variable	Values
Age, years, mean ±SD		63.6 ±20
Sex, n (%)	Male	97 (56.4%)
	Female	75 (43.6%)
Nationality, n (%)	Saudi	143 (83.1%)
	Non-Saudi	29 (16.9%)
Symptoms and vital signs, n (%)	Cough	140 (81.4%)
	Respiratory distress	49 (28.5%)
	Gastrointestinal symptoms	48 (27.9%)
	Low oxygen saturation	54 (31.3%)
	Fever	35 (20.3%)
Comorbid diseases, n (%)	Cardiac	95 (55.2%)
	Pulmonary	46 (26.7%)
	Kidney	30 (17.4%)
	Diabetes	85 (49.4%)
	Obesity	56 (32.6%)
	Immune diseases	2 (1.2%)
	Cancer	11 (6.4%)
	Transplantation history	3 (1.7%)
Number of comorbid diseases, n (%)	None	25 (14.5%)
	1-2	59 (34.3%)
	3-4	67 (39.0%)
	More than 4	21 (12.2%)
Total, n (%)		172 (100.0%)

Table 2 presents the association between patients’ demographics, comorbid diseases, and the need for invasive or noninvasive ventilation. While 58 (33.7%) patients required invasive ventilation, 70 (40.7%) needed noninvasive ventilation. Moreover, most of the patients who needed invasive ventilation were males (n=31, 32,0%), Saudi nationals (n=51, 35.7%), and had a mean age of 65.8 ±19.9 years. Also, most had three to four comorbidities (n=24, 35.8%). No significant association was found between the variables. Most of the patients who needed noninvasive ventilation were females (n=35, 46.7%), of non-Saudi nationality (n=12, 41.4%), and had a mean age of 61.6 ±21 years. Most of them had three to four comorbid diseases (n=30, 44.8%). The mean number of diseases was 2.8 ±1.6. No significant association was found between the variables. Regarding the association between types of comorbid conditions and the need for invasive ventilation, most of the patients who needed invasive ventilation (n=58) had heart diseases (n=36, 37.9%), diabetes (n=32, 37.6%), lung disease (n=15, 32.6%) and obesity (n=18, 32.1%). No significant association between these variables was found. Regarding the association between types of comorbid conditions and the need for noninvasive ventilation, most of the patients who needed noninvasive ventilation (n=70) had heart diseases (n=40, 42.1%), diabetes (n=37, 43.5%), lung disease (n=23, 50.0%), and obesity (n=27, 48.2%). There was no significant association between the variables.

**Table 2:** Association between patient demographics, comorbid diseases, and the need for invasive or noninvasive ventilation (n=172) SD: standard deviation

Variables		Need for invasive ventilation			Need for noninvasive ventilation		
		Yes (n=58)	No (n=114)	P-value	Yes (n=70)	No (n=102)	P-value
Age, years, mean ±SD		65.8 ±19.9	62.5 ±20	0.306	61.6 ±21	65 ±19.2	0.272
Gender, n (%)	Male	31 (32.0%)	66 (68.0%)	0.578	35 (36.1%)	62 (63.9%)	0.161
	Female	27 (36.0%)	48 (64.0%)		35 (46.7%)	40 (53.3%)	
Nationality, n (%)	Saudi	51 (35.7%)	92 (64.3%)	0.231	58 (40.6%)	85 (59.4%)	0.935
	Non-Saudi	7 (24.1%)	22 (75.9%)		12 (41.4%)	17 (58.6%)	
Comorbid diseases, n (%)	Cardiac	36 (37.9%)	59 (62.1%)	0.198	40 (42.1%)	55 (57.9%)	0.676
	Pulmonary	15 (32.6%)	31 (67.4%)	0.852	23 (50.0%)	23 (50.0%)	0.133
	Kidney	14 (46.7%)	16 (53.3%)	0.099	10 (33.3%)	20 (66.7%)	0.366
	Diabetes	32 (37.6%)	53 (62.4%)	0.282	37 (43.5%)	48 (56.5%)	0.455
	Obesity	18 (32.1%)	38 (67.9%)	0.761	27 (48.2%)	29 (51.8%)	0.163
	Immune diseases	1 (50%)	1 (50%)	0.624	1 (50%)	1 (50%)	0.788
	Cancer	3 (27.3%)	8 (72.7%)	0.640	7 (63.6%)	4 (36.4%)	0.109
	Transplantation history	2 (66.7%)	1 (33.3%)	0.223	0 (0%)	3 (100%)	0.148
Number of comorbid diseases, n (%)	None	5 (20.0%)	20 (80.0%)	0.249	5 (20.0%)	20 (80.0%)	0.134
	1-2	19 (32.2%)	40 (67.8%)		27 (45.8%)	32 (54.2%)	
	3-4	24 (35.8%)	43 (64.2%)		30 (44.8%)	37 (55.2%)	
	More than 4	17 (81.0%)	4 (19.0%)		12 (57.1%)	9 (42.9%)	
Number of diseases, mean ±SD		3 ±1.8	2.5 ±1.8	0.060	2.8 ±1.6	2.6 ±1.9	0.387

Table 3 presents the association between patient demographics, comorbid diseases, and mortality rates; most of the patients who died (n=90) were older (mean age: 69.6 ±18.2 years; p<0.001, odds ratio: 1.049, 95% CI: 1.018-1.451). Most of them were males (n=50, 51.5%) and Saudi nationals (n=84, 58.7%; p<0.001). Most of them had three to four comorbid diseases (n=39, 58.2%; p=0.017). The mean number of diseases was 3.1 ±1.7 (p=0.001). Also, significant associations were found between mortality and diabetes, cardiac, and kidney diseases (p=0.009, 0.025, and 0.003 respectively).

**Table 3:** Association between the patient demographics, comorbid diseases, and mortality rate (n=172) SD: standard deviation

Variables		Mortality		P-value
		Yes (n=90)	No (n=82)	
Age, years, mean ±SD		69.6 ±18.2	56.9 ±19.9	<0.001
Gender, n (%)	Male	50 (51.5%)	47 (48.5%)	0.816
	Female	40 (53.3%)	35 (46.7%)	
Nationality, n (%)	Saudi	84 (58.7%)	59 (41.3%)	<0.001
	Non-Saudi	6 (20.7%)	23 (79.3%)	
Comorbid diseases, n (%)	Cardiac	57 (60%)	38 (40%)	0.025
	Pulmonary	24 (52.2%)	22 (47.8%)	0.981
	Kidney	23 (76.7%)	7 (23.3%)	0.003
	Diabetes	53 (62.4%)	32 (37.6%)	0.009
	Obesity	24 (42.9%)	32 (57.1%)	0.084
	Immune diseases	2 (100%)	0 (0%)	0.175
	Cancer	8 (72.7%)	3 (27.3%)	0.161
	Transplantation history	3 (100%)	0 (0%)	0.095
Number of comorbid diseases, n (%)	None	7 (28%)	18 (72%)	0.017
	1-2	29 (49.2%)	30 (50.8%)	
	3-4	39 (58.2%)	28 (41.8%)	
	More than 4	15 (71.4%)	6 (28.6%)	
Number of diseases, mean ±SD		3.1 ±1.7	2.2 ±1.8	0.001

Table 4 presents the association between comorbid diseases and the duration of hospital stay among the patients. Patients with immune disorders and kidney diseases had the most prolonged hospital stay (28.5 ±31.8 and 17.1 ±12.8 days respectively) and this association was significant (p=0.048 and 0.010 respectively), while patients with obesity (10.6 ±10.5 days) and cancer (6.1 ±5.9 days) had the shortest duration of hospital stay.

**Table 4:** Association between comorbid diseases and the duration of hospital stay among the patients (n=172) SD: standard deviation

Diseases	Hospital stay, days, mean ±SD	P-value
Cardiac	14.2 ±13.6	0.599
Pulmonary	14.6 ±15.4	0.149
Kidney	17.1 ±12.8	0.010
Diabetes	13 ±11.8	0.813
Obesity	10.6 ±10.5	0.769
Immune diseases	28.5 ±31.8	0.048
Cancer	6.1 ±5.9	0.802
Transplantation history	25.3 ±23.5	0.161

## Discussion

The COVID-19 pandemic has rapidly evolved across the world. The demographic distribution and temporal trends in adverse clinical outcomes associated with the condition, such as ICU admissions, ARDS/respiratory failure, IMV, and all-cause mortality, must be understood on a global scale [4]. In this cross-sectional study, we reviewed the medical records of 172 adult patients to assess the clinical characteristics and outcomes of COVID-19 ICU patients in the Qassim Region, Saudi Arabia. Our study findings showed that the patients were mostly male (n=97, 56.4%), with a mean age of 63.6 ±20 years. A similar result was observed in another study where COVID-19 was most commonly found in adult male patients aged 34-59 years [11]. Another study involving 22 COVID-19 ICU patients reported a median age of 54.5 years, and 16 (72.7%) of the patients were males. Also, all of them had at least one comorbidity [12].

In our study, most of the patients had cardiac diseases (n=95, 55.2%), diabetes (n=85, 49.4%), and obesity (n=56, 32.6%). In another study, the most common comorbidity among patients was diabetes mellitus (n=13, 13.6%), followed by hypertension (n=11, 10.9%), and asthma (n=3, 3.5%) [11]. Moreover, another study reported comorbidities in 220 (20.1%) patients, with hypertension in 97 (8.8%) cases and diabetes mellitus in 83 (7.6%) [13]. Our data are also in agreement with findings from an observational study that suggests that chronic conditions are associated with an increased risk of a more severe infection requiring hospitalization among COVID-19 patients [14].

This study reported the clinical characteristics of and outcomes in patients who needed mechanical/non-mechanical ventilation; 58 (33.7%) patients needed mechanical (invasive) ventilation and 31 (32.0%) of them were males. Most of them had a heart disease (n=36, 37.9%). Of note, 70 (40.7%) patients in our study needed non-mechanical (noninvasive) ventilation; most of them were female (n=35, 46.7%) and had a heart disease (n=40, 42.1%). According to a recent study, non-survivors used interventional invasive mechanical ventilation at a much higher rate than survivors (83% vs. 31%, p=0.001) [7]. Among survivors, the period of mechanical ventilation was significantly shorter (6.3 vs. 12.2 days) with 270 (31.5%) patients requiring it throughout their hospital stay [7]. Another study found a lower mortality rate than our study and documented survivors who underwent prolonged mechanical ventilation for more than three weeks [8]. In a Canadian study, among patients admitted to the ICU for COVID-19, about two-thirds received invasive mechanical ventilation and one-quarter died [15].

The results of our study showed that COVID-19 has a more unfavorable impact on elderly patients with comorbid diseases. Most of the patients who died (n=90, 52.3%) were elderly (mean age: 69.6 ±18.2 years, p<0.001), suggesting elevated risk throughout the course of the disease among older patients. These results are in accordance with findings from other prior studies. Elderly patients had a higher prevalence of pre-existing comorbidities, which could have negatively affected patients’ ability to fight against infections [16]. In addition, Takahashi et al. [14] found that declined T-cell response is associated with advanced age, which could lead to lower efficacy in viral clearance and a higher likelihood of inflammatory cytokine storm, resulting in poor health outcomes.

With regard to the association between comorbidities and death, most of the patients who died in our study had heart disease (n=57, 60.0%) or diabetes (n=53, 62.4%). Another study conducted in Saudi Arabia reported that the overall death rate among COVID-19 patients was 614 (41.8%), with a much higher rate among diabetics (n=341, 55.5%), hypertensive patients (n=309, 50.3%), and patients with ischemic heart disease (n=99, 16.1%) [7].

In our study, most of the patients who had a long hospital stay had immune diseases (mean number of days: 28.5 +31.8, p=0.048), and kidney diseases (mean number of days: 17.1 ±12.8, p=0.010), while patients with obesity (10.6 ±10.5 days) and cancer (6.1 ±5.9 days) had the shortest duration of hospital stay. The findings of another study revealed a longer hospital stay, longer time in the ICU, a higher probability of ICU admission, and a more urgent need for mechanical ventilation among patients with more chronic conditions compared to other groups [12].

Several limitations should be taken into consideration when interpreting the results of this study. We employed a retrospective, short-term study design; a prospective research design would have given us greater insight into the outcomes of severe COVID-19 infections admitted to the ICU. The study was also limited by its relatively small sample size.

## Conclusions

COVID-19 infection is associated with a wide range of characteristics and outcomes. Based on our findings, most of the COVID-19 ICU patients were elderly males with multiple comorbidities, mainly diabetes, cardiac diseases, and obesity. A significant association was found between certain comorbidities and death rates in our patients, as well as between specific comorbidities and the duration of hospital stay. Raising awareness about risk factors of COVID-19 infection will improve the clinical outcomes among patients by ensuring timely and correct resource allocations as well as the implementation of appropriate preventive measures. Further studies are required to validate our findings.
